# Is It Time to Expand Newborn Screening for Congenital Hypothyroidism to Other Rare Thyroid Diseases?

**DOI:** 10.3390/ijns11030065

**Published:** 2025-08-20

**Authors:** Antonella Olivieri, Maria Cristina Vigone, Mariacarolina Salerno, Luca Persani

**Affiliations:** 1 Department of Cardiovascular and Endocrine-Metabolic Diseases and Aging, Italian National Institute of Health, 00161 Rome, Italy; 2 Department of Pediatrics, IRCCS San Raffaele Scientific Institute, 20132 Milan, Italy; vigone.mariacristina@hsr.it; 3 Pediatric Endocrinology Unit, Department of Translational Medical Sciences, University of Naples Federico II, 80131 Naples, Italy; salerno@unina.it; 4 Department of Medical Biotechnology and Translational Medicine, University of Milan, 20133 Milan, Italy; luca.persani@unimi.it; 5 Department of Endocrine and Metabolic Diseases, IRCCS Istituto Auxologico Italiano, 20149 Milan, Italy

**Keywords:** congenital hypothyroidism, newborn screening, rare diseases

## Abstract

Congenital hypothyroidism (CH) is a heterogeneous condition present at birth, resulting in severe-to-mild thyroid hormone deficiency. This condition is difficult to recognize shortly after birth. Therefore, many countries worldwide have implemented newborn screening (NBS) programs for CH since the 1970s. The most recent European guidelines strongly recommend screening for primary CH, as well as for central CH when financial resources are available. However, no consensus has been reached yet to screen more rare forms of CH, such as Allan–Herndon–Dudley syndrome (AHDS), an X-linked condition linked to mutations in the gene encoding a transmembrane monocarboxylate transporter (MCT8), resistance to thyroid hormone beta (RTHβ), and resistance to thyroid hormone alfa (RTHα). The combined measurement of thyroid-stimulating hormone (TSH) and total thyroxine (TT4) on DBS currently allows the recognition of central CH (TSH low/normal and low TT4 without defects in transport proteins). With the introduction of liquid chromatography coupled to tandem mass spectrometry (LC-MS/MS) for measurement of free triiodothyronine (FT3) and free thyroxine (FT4), it would be possible to screen for RTHβ (TSH normal/high and high FT4). More complicated would be the method to screen RTHα. It would require the combined measurement of FT4 and FT3 and the determination of FT3/FT4 ratio, while the combined measurement of FT3 and reverse T3 (rT3) to calculate FT3/rT3 ratio would be useful to screen AHDS. In this article, we provide some reflections on expanding NBS for primary CH also to other rare forms of CH.

## 1. Introduction

Congenital hypothyroidism (CH) is a heterogeneous condition present at birth, resulting in severe-to-mild thyroid hormone deficiency. CH may be caused by abnormal development or function of thyroid gland (primary CH), or by dysfunction of hypothalamus or pituitary gland (central CH), or more rarely by impaired transport or action of thyroid hormone at the tissue level, such as monocarboxylate transporter 8 defects or Alan–Herndon–Dudley syndrome, resistance to thyroid hormone alfa (RTHα) or resistance to thyroid hormone beta (RTHβ) [1,2,3]. Thyroid hormone deficiency early in life is harmful for growth and neurocognitive development. The critical period for brain development occurs in the first three years of life, during which thyroid hormones play a key role.

Thyroid hormone deficiency is difficult to recognize shortly after birth. Therefore, many countries worldwide have implemented newborn screening (NBS) programs for CH since the 1970s [4,5,6]. The most recent European guidelines strongly recommend screening for primary CH. These guidelines also recommend screening for central CH when financial resources are available. However, no consensus has been reached yet to screen more rare forms of CH [1].

Here we present the Italian NBS program for CH as an example of longstanding, nationwide screening program for the disease and provide some considerations on the possibility of expanding this program to more rare forms of congenital thyroid diseases.

## 2. Italian NBS Program for CH

In Italy, a nationwide mandatory program of NBS for the identification and early treatment of CH, phenylketonuria, and cystic fibrosis was established in 1992 [7]. Since then, around 16 million infants have been screened in our country. More recently, an additional nationwide NBS program aimed at identifying more than 50 rare inherited metabolic diseases has been introduced [8]. This new program has reduced the window of age at screening to 48–72 h of life. The Italian NBS program for CH is supported by a nationwide surveillance system carried out by the Italian National Registry of Infants with CH (INRICH) at the Italian National Institute of Health [9].

Currently, 16 regional or inter-regional screening laboratories are active in our country to screen primary CH. Since measurement of TSH is the most sensitive test for detecting primary CH and according to the European guidelines [1], all the Italian laboratories use the measurement of TSH on dried blood spot (DBS) as the primary screening test to identify all forms of primary CH (mild, moderate, and severe). In all the laboratories, the measurement of TSH is performed by means of GSP Neonatal hTSH Time-Resolved Fluorescence assay (Revvity, Turku, Finland). A re-screening on DBS at 2 weeks of life is also performed in infants at risk of delayed TSH rise, i.e., infants with gestational age <37 weeks’ gestation and/or low birth weight, with extra-thyroidal malformations, admitted to NICU, twins.

On first screening, the TSH threshold values used for referral (after confirmation of the screening test results) are variable, ranging 6–10 mIU/L blood according to the percentile of the TSH distribution chosen in each region (INRICH data). However, if the screening test is ≥40 mIU/L blood, the infant is immediately referred to the pediatric clinical center without waiting for confirmatory laboratory test, as this value is highly suggestive of moderate-to-severe primary CH [1]. Only in the Campania region is the TSH threshold for an immediate referral to the pediatric clinical center ≥20 mIU/L blood. On re-screening (2 weeks of life), the TSH threshold levels are also variable, ranging 3–9 mIU/L blood (INRICH data).

Over the years, the increased assay sensitivity and a more effective analysis of the distribution of TSH in the screened neonatal population have led to lower TSH cutoffs in some screening programs, including the Italian NBS program for CH [10].

Lowering of TSH screening cutoffs along with other factors, such as increased survival rate of preterm babies and twin births, changes in screening population demographics, and a progressive adoption of re-screening strategy in infants at risk of delayed TSH rise [11,12,13,14,15,16,17], have led to an increased incidence of primary CH. Currently in Italy, the estimated incidence of primary CH is 1:1100 live births (www.iss.it/rnic accessed on 18th August 2025). This incidence is similar to that recently reported in other countries using similar NBS strategies for CH [18,19,20,21].

The most recent European guidelines recommend adding measurement of total (TT4) or free T4 (FT4) to TSH to screen for central CH [1]. At present in Italy, only 3 regions (Campania, Calabria, Sardinia) have adopted a screening strategy employing TSH + TT4 testing to detect both primary and central CH. TT4 is measured by means of GSP Neonatal hT4 Time-Resolved Fluorescence assay. In these regions, the blood TSH threshold values used on the first screening are set at the 97.5th percentile of the distribution, corresponding to 6.0 mIU/L in Campania, 7.0 mIU/L in Calabria, and 5.5 mIU/L in the Sardinia region; whereas the blood TT4 threshold value is set at the 0.15th percentile in Campania (4.2 μg/dL), the 2.5th percentile in Calabria (7.0 μg/dL), and the 2.2nd percentile in the Sardinia region (6.0 μg/dL). On re-screening (2 weeks of life), the blood TSH threshold values for referral are 5.0 mIU/L in Campania, 3.0 mIU/L in Calabria, and 5.5 mIU/L in the Sardinia region, while the blood TT4 threshold is ≤4.0 μg/dL in all the three regions. The risk of false-positive results, deriving from preterm and sick neonates often showing low TT4 with non-elevated TSH levels [22], is overcome by the re-screening procedure at 2 weeks of age.

It is evident that these regional screening programs testing for both primary and central CH are expensive, and an optimization process is necessary to make these programs cost-effective in terms of costs and workload. This process could include the introduction of the routinary measurement of thyroxine-binding globulin (TBG) with the use of specific reference intervals to rule out cases of complete and partial TBG deficit [6].

Although epidemiological data on the incidence of central CH in these regions are not yet available, it can be assumed that the diagnosis of central CH may be delayed in the other Italian regions.

## 3. Central CH

Central CH results from a dysfunction of the hypothalamus or pituitary gland, leading to inadequate stimulation of a normal thyroid. In comparison with primary CH, central CH can be more difficult to diagnose because it does not present with elevated TSH levels, resulting in the main “false-negative” condition by the TSH screening test. The diagnosis of central CH is most often based on finding low or normal serum FT4 associated with an inappropriately low or normal serum TSH concentration. It is important to be aware of confounding factors and other conditions that may present with a similar biochemical picture (e.g., TBG defects or Non-Thyroidal Illness Syndrome, etc.) [23].

Central CH is mainly diagnosed in patients with hypothalamic–pituitary disorders, usually in combination with other pituitary hormone defects. In these cases, the diagnosis of central hypothyroidism is facilitated by the presence of symptoms and signs that are typical of other hormone deficiencies (severe hypoglycemia, presence of micropenis, etc.), whereas the isolated presentation of central CH is difficult to diagnose, because of frequently mild manifestations. Approximately 90 percent of cases of isolated central congenital CH are due to genetic defects. Depending on the cause of the disease, the TSH secretion deficit may be quantitative (reduced number of TSH-secreting cells) or qualitative (altered bioactivity of secreted TSH) [23,24,25].

Congenital forms of central hypothyroidism mostly result from structural lesions such as pituitary hypoplasia, Rathke’s pouch cysts, or midline defects, which mainly cause quantitative defects in TSH secretion. However, functional defects in TSH biosynthesis and/or release are caused by loss-of-function mutations in genes encoding various types of genes, such as *TRHR, TSHβ*, *IGSF1, TBL1X,* or *IRS4* as candidates for isolated central CH or *POU1F1, PROP1, HESX1, LHX3, LHX4, SOX2, SOX3, OTX2*, and *LEPR* as candidates for combined pituitary hormone defects (CPHDs) [1,2,23,26,27]. The mode of inheritance of these defects is variable (autosomal recessive or X-linked inheritance in most cases, but dominant inheritance was described in rare cases of combined pituitary hormone defects).

The reported incidence of central CH detected by neonatal screening varies between 1:30,000 and 1:16,000, depending on the adopted screening strategy [28,29,30]. Early diagnosis of central CH is particularly crucial for growth and neurocognitive development because of the significant impact of thyroid hormone deficiency. Infants with undiagnosed and untreated central CH are at high risk of severe neurodevelopmental delays, including cognitive impairment, motor disabilities, and an overall reduced quality of life. Therefore, as with primary CH, neonatal screening for central CH can also allow an early diagnosis and hence the early initiation of treatment with levothyroxine, which may improve the neurological outcome [2,31].

Combined measurement of TSH and T4 levels in newborn screening can help effectively identify central CH. Early diagnosis not only improves individual health outcomes but also has wider socio-economic benefits. By preventing severe intellectual disability and other health complications, early treatment reduces the long-term healthcare costs associated with managing untreated central CH. Families benefit from reduced emotional and financial burden, and society benefits from the improved productivity and quality of life of individuals who receive timely and appropriate treatment. In addition, the neonatal diagnosis of central hypothyroidism is also effective in making an earlier diagnosis and treatment of multiple pituitary hormone deficiencies, commonly associated with central CH, thus preventing life-threatening conditions [32,33].

Screening for central CH is complicated because the measurement of T4 is influenced by several factors including binding proteins, the clinical condition of the patient, gestational age, and the individual setpoint of the hypothalamic–pituitary–thyroid (HPT) axis [6]. The Netherlands provides an effective newborn screening strategy for central CH. Since 1995, this program has consisted of a three-step approach, with T4 measurement in all newborns as the first step, TSH measurement in the lowest 20% of T4 concentrations, and thyroxine-binding globulin (TBG) measurement in the lowest 5% to prevent false-positive results due to TBG deficiency [6,31]. In this screening program, the calculation of T4/TBG ratio, as an indirect measure for FT4, has effectively lowered the number of false-positive referrals essentially due to infants with complete TBG deficiency. In addition, reference intervals (RIs) for the NBS parameters—total T4, TSH, TBG, and total T4/TBG ratio—have been recently established in the Dutch NBS, so that the use of the TBG reference intervals to identify partial TBG deficiency has further reduced false-positive referrals by approximately 50%. As a result, the Dutch screening algorithm has been adapted to exclude total and partial TBG deficiency (TBG < 105 nmol/L blood) and this led to an improvement in the positive predicted value (currently 21%) while maintaining the current sensitivity of central CH detection [34,35]. It is worth noting that the current three-step T4-reflex TSH-reflex TBG NBS program also led to an improved detection of central CH with an incidence of 1:16,404, which appears to be much higher than that reported in countries using T4-reflex TSH or TSH-based strategies [32]. Regarding primary CH, it is important to underline that this three-step Dutch NBS program effectively detects severe forms of primary CH, with a positive predictive value (PPV) of 55% [34]. Nevertheless, as neonates at risk of primary CH are preselected based on their T4 concentrations (80% of all neonates do not receive a TSH measurement), mild primary CH cases with normal T4 concentrations might be missed. This could contribute to the lower incidence of primary CH in the Netherlands compared to other countries [5,34,35,36].

In Italy, the lack of a uniform NBS program for central CH may lead to delayed diagnosis in different regions, highlighting the need for a nationwide implementation of comprehensive screening protocols. The strategy of using TSH + T4 as primary screening tests, along with TBG measurement and adoption of specific reference intervals, appears to be the most effective strategy to adopt in our country for an effective detection of both primary and central CH on a large scale and at an acceptable cost for our health system. It would eliminate regional disparities and ensure that all newborns with central CH have equal access to early diagnosis and treatment.

## 4. Other Rare Congenital Thyroid Diseases

### 4.1. MCT8 Defects

Allan–Herndon–Dudley syndrome (AHDS) is an X-linked condition prevalently affecting boys, which is associated with severe intellectual and motor disabilities [37]. AHDS was described more than 60 years ago, but only in 2004 was this disease linked to mutations in *SLC16A2*, the gene encoding the transmembrane monocarboxylate transporter 8 (MCT8). This gene is crucial in the transport of the thyroid hormones triiodothyronine (T3) and thyroxine (T4) into several tissues, including the brain [38,39,40]. The estimated incidence of MCT8 defects is around 1:70,000 newborns [38,39]. However, this incidence may be underestimated, and many cases are left underdiagnosed due to the current absence of a recognizable biochemical signature.

As shown in Table 1, the disease is biochemically characterized by high levels of serum free T3 (FT3), low or borderline low levels of serum FT4, low serum reverse T3 (rT3), and a TSH concentration in the normal range [41]. Although these biochemical findings are suggestive of a thyroid hormone disturbance, the clinical presentation observed in AHDS includes both “thyrotoxic” peripheral tissues (bone, heart, skeletal muscles, liver) and the “hypothyroid” brain [37]. Interestingly, the degree of psychomotor impairment is similar or even worse compared to that seen in untreated primary CH. Clinical trials have been recently conducted to understand the efficacy and safety of treatment with a triiodothyronine analog named TRIAC (triiodothyroacetic acid) [42]. This treatment is revealed to be effective in improving peripheral metabolic conditions. Nevertheless, it has almost no effect on psychoneuromotor deficits because of the delayed start of treatment. In this regard, a recent study has shown that prenatal intraamniotic thyroxine treatment improved the neuromotor and neurocognitive function in a boy with MCT8 deficiency born to a carrier mother, thus showing that prenatal start of treatment with thyroid hormone analogs can rescue at least part of the phenotype [43].

### 4.2. Resistance to Thyroid Hormone Beta

The key characteristic of RTHβ is a combination of raised thyroid hormones with non-suppressed TSH (Table 1) [44]. RTHβ patients can exhibit features of hyperthyroidism or hypothyroidism, reflecting either compensated hormone resistance in TRβ-expressing tissues (e.g., liver, pituitary) or approximately normal sensitivity to high circulating thyroid hormones in TRβ-expressing tissues (e.g., heart, brain). The condition is dominantly inherited and co-segregates with dominant negative heterozygous mutations in *THRB*, the gene encoding for thyroid receptor β (TRβ) [45]. Neurocognitive manifestations of RTHβ are anxiety and sleep disturbance, attention-deficit hyperactivity disorder [46,47], variable intellectual disability (lower nonverbal intelligence), language difficulties [48], and poor educational outcome [49]. Severe intellectual disability and cochlear dysfunction are characteristic of homozygous cases [50,51]. Recently reported data show that RTHβ patients are at significantly higher risk of major cardiovascular events (atrial fibrillation, myocardial infarction, heart failure) and of earlier mortality [52,53]. Inhibition of TSH secretion into lower circulating T4 and T3 obtained by treatment with TRIAC appears efficient in controlling thyrotoxic manifestations in adults and children with RTHβ [54,55]. An improvement in some neurological manifestations has also been observed, but clinical trials are required to understand if early (neonatal) start of TRIAC treatment can rescue the neurocognitive manifestations and/or reduce the adverse cardiovascular manifestations of the disease [54,56,57]. The incidence of RTHβ has been estimated to range between 1:40,000 and 1:19,000 neonates in the USA and Spain, respectively, by the finding from neonatal screening of raised total T4 associated with normal TSH levels [58,59].

### 4.3. Resistance to Thyroid Hormone Alfa

RTHα is a rare disorder, with less than 100 affected individuals reported to date. Although the phenotype is highly variable, many patients exhibit similar clinical features, including psychomotor delay (cognitive impairment, delayed growth milestones, dyspraxia) and peripheral manifestations. The RTHα phenotype recapitulates most of the features of untreated congenital hypothyroidism except the typical biochemical signature. The thyroid function tests are similar to those seen in AHDS or central hypothyroidism: TSH is generally within the reference range (rarely borderline elevated), FT4 is borderline low, and FT3 is borderline high, and this is hampering the diagnostic possibility of this condition (Table 1).

The disease is caused by dominant negative heterozygous mutations in *THRA*, the gene encoding for TRα [60,61]. The identified mutations lie in the same hot spots in the T3-binding domain of the receptor and the molecular mechanisms underlying this disease are similar to those previously described for RTHβ. To date, levothyroxine is the main treatment described for RTHα [62,63].

Although data are restricted to case reports or case series, thyroxine treatment in RTHα seems safe and well tolerated and provides beneficial effects for most patients [63,64,65]. The therapeutic responses are variable, depending upon the degree of resistance generated by the mutation and the age of treatment start. The neurocognitive outcome can be predicted to significantly improve or even normalize if the treatment could be started in the neonatal period (or even prenatally) for patients with mutant receptors that have a diminished, but not abolished, T3-binding affinity. The frequency of RTHα is unknown and underestimated at present because of the lack of a clear-cut biochemical signature, but its incidence can be expected to be similar to that of RTHβ, since both diseases are caused by dominant negative variants occurring in the same hot spots within the T3-binding domains, which are highly conserved between the two receptors.

## 5. NBS for Rare Congenital Thyroid Diseases

The early recognition of these rare congenital thyroid diseases would allow the neonatal start of thyroid hormone treatment aiming to rescue, at least partially, the neurocognitive and developmental phenotypes of these dramatic diseases. Particularly, the combined measurement of TSH and TT4 on DBS would easily allow the recognition of central CH (TSH low/normal and low TT4 without defects in transport proteins) and RTHβ (TSH normal/high and high FT4). More complicated would be the method to screen RTHα and AHDS. This would require the combined measurement of FT3 and FT4 in DBS samples with the calculation of FT3/FT4 ratio (low) to detect cases of RTHα, while the combined measurement of FT3 and rT3 with the calculation of FT3/rT3 ratio (high) could be used to detect infants with AHDS (MCT8 deficiency).

It is well known that the main drawback in the determination of circulating thyroid hormones is the interference of defects in transport proteins [66]. To avoid any interfering effect, the measurement of free thyroid hormones using equilibrium dialysis or ultrafiltration combined with liquid chromatography coupled to tandem mass spectrometry (LC-MS/MS) is considered the most suitable approach for measurement of concentration of free thyroid hormones T3, T4, and rT3 [41,67]. Although LC-MS/MS technology is generally employed for second-tier tests to reduce the false-positive rate, in many countries it is currently applied to primary screening for some inherited metabolic diseases for which other methods, including genomic screening, are not adequate [68]. The measurement of thyroid hormones by means of LC-MS/MS is therefore feasible. We are confident that with reduction in cost of LC-MS/MS measurements, the routinary application of this technology to screen for rare congenital thyroid diseases will be possible in a reasonable time.

In countries where a NBS program for primary CH is active, early diagnosis and treatment have dramatically changed the life of affected children, avoiding severe neurocognitive sequelae of a disease that is still the most frequent endocrine disease in infancy, with a case in every 1100–1500 liveborns [5]. Central CH and resistance to thyroid hormones RTHα and RTHβ are more rare forms of congenital hypothyroidism but similarly harmful without an early diagnosis and treatment.

In 1968, Wilson and Jungner criteria to screen for a disease included the high frequency in the population of the disease to screen, as well as the availability of treatment for patients with a disease recognized early by screening [69]. The introduction of the expanded NBS for several metabolic rare diseases in Italy, as in other countries, has led to reconsidering these criteria, introducing the new concept that screening should identify *actionable* diseases, including treatable diseases. Actionable diseases are conditions where early interventions lead to health gain for newborns and where early diagnosis avoids the lengthy diagnostic odyssey [70]. This therefore implies that, although the incidence of some of these disorders is very low (<1:100,000) and some of them are untreatable, their early detection and treatment is nevertheless beneficial because they can prevent the onset of disease symptoms or delay disease progression, improving the quality of life of the newborn, their families, and society [70]. These new concepts can be easily applied to central CH, MCT8 defects, RTHα, and RTHβ as well.

In addition, the development of new technologies such as tandem mass spectrometry, which offers the possibility of screening for many conditions using a single DBS [71,72,73,74], makes feasible the measurement of FT4, FT3, and rT3 on DBS with the possibility of providing early diagnosis and preventing or ameliorating the long-term consequences of the rare congenital forms of hypothyroidism.

Next-generation sequencing (NGS) may also be a powerful tool for diagnosing rare congenital thyroid diseases. Its ability to analyze multiple genes simultaneously and identify novel mutations makes it a powerful approach for improving diagnosis, treatment, and research in the field of rare congenital thyroid diseases [75].

## 6. Conclusions

We believe that the current Italian NBS program, which is successful in screening for primary CH, could be expanded to address regional disparities and to detect the broad spectrum of other rare congenital thyroid disorders.

Although carrying out such a comprehensive screening program would generate additional costs in terms of laboratory and clinical activities, a uniform national protocol where the measurement of FT3, FT4, and rT3 is added to TSH by means of LC-MS/MS could eliminate regional disparities and ensure that all newborns, including those with rare congenital thyroid diseases, have equal access to early diagnosis and treatment, improving individual health outcomes, and reducing long-term healthcare costs as well as the burden on families.

Expanding NBS programs for CH to include other rare congenital thyroid disorders is therefore a crucial step towards giving all newborns the best start in life. Early detection and intervention are key to preventing serious health problems and improving the overall quality of life of affected children. For this reason, further studies are necessary to discover new and more specific biomarkers that, along with TSH FT3, FT4, rT3, as well as NGS (comprehensive targeted NGS panels up to whole-genome screening), can help to develop an effective expanded NBS program for rare congenital thyroid diseases.

## Figures and Tables

**Table 1:** Molecular and biochemical signature, treatment, and screening tests in rare congenital thyroid disorders.

Molecular and Biochemical Signature and Treatment	Central Hypothyroidism	Resistance to Thyroid Hormone Beta (RTHβ)	Resistance to Thyroid Hormone Alpha (RTHα)	Monocarboxylate Transporter 8 (MCT8) Deficiency
Gene	*several*	*THRB*	*THRA*	*SLC16A2*
Inheritance pattern	Variable *	Dominant	Dominant	X-linked
Serum Free T4	low	high	low-normal or low	low-normal or low
Serum Free T3	low or normal	high	high-normal or high	high or high-normal
Serum Reverse T3	--	--	--	low
Serum TSH	low or normal (rarely, mildly raised)	normal or high	normal (rarely, mildly raised)	normal (rarely, mildly raised)
Treatment	LT4	TRIAC	LT4	TRIAC
**Screening tests on DBS and** **expected results**				
Current Screening tests	TSH + TT4	--	--	--
Possible future screening tests **		TSH + FT4	TSH, FT4 + FT3, and calculation of FT3/FT4 ratio	TSH, FT3, rT3, and calculation of FT3/rT3 ratio
Expected results of screening tests	low/normal TSH and low TT4	normal/high TSH and high FT4	low FT3/FT4 ratio	high FT3/rT3 ratio

* List of candidate genes for central CH: TRHR (recessive), TSHβ (recessive), IGSF1 (X-linked), TBL1X (X-linked), IRS4 (X-linked), LEPR (recessive), POU1F1 (recessive or dominant), PROP1 (recessive), HESX1 (recessive or dominant), LHX3 (recessive), LHX4 (recessive or dominant), SOX2 (dominant), SOX3 (X-linked), OTX2 (dominant). ** Measurement of FT3, FT4, and rT3 performed by means of LC-MS/MS.

## Data Availability

Not applicable.

## Abbreviations

The following abbreviations are used in this manuscript:

AHDS	Alan–Herndon–Dudley Syndrome
CH	Congenital Hypothyroidism
FT4	Free Thyroxine
FT3	Free Triiodothyronine
MCT8	Transmembrane monocarboxylate transporter 8
NBS	Newborn Screening
RTHα	Resistance to Thyroid Hormone alfa
RTHβ	Resistance to Thyroid Hormone beta
TRIAC	Triiodothyroacetic acid
TSH	Thyroid-Stimulating Hormone
TT4	Total Thyroxine
T3	Triiodothyronine
